# Ethnic-Cultural Bullying Versus Personal Bullying: Specificity and Measurement of Discriminatory Aggression and Victimization Among Adolescents

**DOI:** 10.3389/fpsyg.2019.00046

**Published:** 2019-02-01

**Authors:** Antonio J. Rodríguez-Hidalgo, Juan Calmaestra, José A. Casas, Rosario Ortega-Ruiz

**Affiliations:** Department of Psychology, Universidad de Córdoba, Córdoba, Spain

**Keywords:** bullying, discrimination, peer victimization, ethnic-cultural, racism, validation, questionnaire

## Abstract

The present study contrasts personal bullying with ethnic-cultural bullying. A representative pluricultural sample from a Spanish adolescent population of Secondary Education took part in the study (*N* = 27369). The sample filled in the EBIPQ to measure personal bullying. Additionally, they filled in an adaptation of this questionnaire to measure the ethnic-cultural bullying: the EBIPQ-ECD. The EBIPQ-ECD validation showed optimal psychometric properties and a bidimensional structure: ethnic-cultural victimization and ethnic-cultural aggression. The same roles of participation in personal bullying —aggressor, victim, bully/victim, non-involved— were observed in ethnic-cultural bullying, but they did not coincide with each other in a considerable part. Therefore, we concluded that ethnic-cultural bullying is a different phenomenon from personal bullying, with the possibility of certain dynamism existing between both. To prevent and mitigate ethnic-cultural bullying, educational inferences are proposed. We also recommend the use of the EBIPQ-ECD as a tool to evaluate and detect ethnic-cultural aggressions and victimization.

## Introduction

Ethnic-cultural discrimination in schools is a phenomenon documented in different countries that seems to threaten personal and social development from early ages and that sometimes even affects the health of students whose family origin or culture differs from the majority (Hooks and Miskovic, 2010; McKinney, 2010; Zembylas, 2010; Zinga and Gordon, 2014; Brüggemann and D’Arcy, 2017; Gkofa, 2017). In adolescents from minority cultural groups, the perception of social discrimination predicts mistrust toward the cultural majority (Benner and Graham, 2013). When students from minority groups experience ethnic-cultural discrimination at school, they lower their expectations on what school can offer them (Cooper and Sánchez, 2016). Discrimination is very much related to loneliness and depression (Priest et al., 2014).

One part of the discrimination is produced by exposure to *microaggressions* in social contexts, which, like school, are for teaching and development. It refers to subtle aggressive behaviors toward minorities based on racial or ethnic-cultural prejudices (Dávila, 2015). It sometimes involves messages that teachers issue on low academic or progress expectations, and other times, disqualifications of customs or attitudes that they attribute to the cultural group. These microaggressions act as an educational barrier, with effects not only on the peers’ discriminatory attitudes but also on academic performance (Baysu et al., 2016), and predict the psychological imbalance of the high-schoolers that suffer them (Benner and Graham, 2013). In relationships among peers, the concurrence of bullying and discrimination is specially worrying. It is known that high-schoolers that suffer bullying and high racial discrimination present a higher probability of suicidal ideas than those who suffer high racial discrimination but not bullying (Garnett et al., 2014).

The present research focuses on peer aggressions and victimization, specifically in the intersection between bullying as a phenomenon that afflicts a significant number of high-schoolers —to which we will call personal bullying— and ethnic-cultural discrimination that we think is frequently found in pluricultural schools. This research consists of a contrast study between the general phenomenon of bullying and the specific phenomenon of ethnic-cultural bullying or cultural, racial or xenophobic discrimination, the purpose of which is to identify whether the latter has a distinctive prominence over the former.

Bullying among students occurs when one or several students develop aggressive behaviors toward another student, whom they mean to harm repeatedly, in an interpersonal relationship of a real or imaginary imbalance of power or strength (Olweus, 1996, 2013). The bullying aggressor forces the victim by means of physical or psychological damage, who feels more and more defenseless and unable to escape from the situation: *victimization* (Sentse et al., 2017). Some studies indicate that we find higher levels of bullying — not only among different ethnic-cultural groups but also within each group — in contexts where there is more ethnic-cultural diversity (e.g., Tolsma et al., 2013). Nevertheless, other studies show that the ethnic-cultural composition cannot be associated with the perpetration of bullying, and that the high presence of other minorities could act as a damper of peer victimization (e.g., Vitoroulis et al., 2016).

In recent years, some studies have shown that the members of minority ethnic-cultural groups are more likely to be involved in bullying than the members of majority groups, as either aggressors (Tippett et al., 2013; Tolsma et al., 2013), victims (Strohmeier et al., 2011; Stefanek et al., 2012; Goldweber et al., 2013; Sulkowski et al., 2014; Llorent et al., 2016) or as bully/victims (Goldweber et al., 2013). In contrast, other studies conclude that there are no significant differences when comparing ethnic-cultural minorities with the majority with regards to being a victim of bullying (Durkin et al., 2012; Rodríguez-Hidalgo et al., 2014; Vitoroulis and Vaillancourt, 2015). Despite these differences of opinion, a lot of studies concur that one of the most frequent attributions of bullying victims as the target of aggressions by peers is being different (Fluck, 2017). There are many differences that are used among minors in order to attack in an unfair, racist or xenophobic way: to have a different appearance, to come from a different country, to have a different skin color, to belong to a different religion, to belong to a distinct cultural subgroup or class, to have idiomatic difficulties or to have a different social status among cultural groups (e.g., Eslea and Mukhtar, 2000; Mendez et al., 2012; Sulkowski et al., 2014). When racial interpersonal behaviors occur, preexisting stereotypes are activated in the aggressor when facing phenotypical or cultural characteristics of the individual who is subjected to it, what influences on the perception and behavior the first one has on the other (Wout et al., 2010).

In the last three decades, the study of bullying in relation to ethnic-cultural differences has changed from collecting some evidence on the existence of racial victimization among high-schoolers in some general studies on bullying (e.g., Lloyd and Stead, 2001; Blaya et al., 2006) to develop two lines of study: (a) a line which considers peer victimization caused by multiple ways of interpersonal aggression, including ethnic-cultural discriminatory forms (e.g., Collins et al., 2004; Strohmeier et al., 2011); (b) another line which in parallel considers *personal victimization*, caused by aggressions that are not specifically ethnic-cultural, and *ethnic-cultural victimization*, caused by ethnic-cultural aggressions (e.g., Verkuyten and Thijs, 2002, 2006; McKenney et al., 2006; Monks et al., 2008).

Ethnic-cultural victimization, like personal victimization, has a structure of *multivictimization*: different ways of aggression — physical, verbal and relational aggression, both direct and indirect — may cause damage to the victim (Rodríguez-Hidalgo et al., 2015; Baysu et al., 2016). The ways in which personal aggression and ethnic-cultural aggression can be expressed are similar, differentiating each other in the aggressor’s explicit declaration during the aggression or in the implicit motivation perceived by the victim: *personal* versus *ethnic-cultural* (Monks et al., 2008). Scientific studies indicate that personal multivictimization influences the students regardless of their ethnic-cultural group, whilst ethnic-cultural multivictimization affects certain students from some minority cultural groups more (e.g., Rodríguez-Hidalgo et al., 2014).

Boys and girls who recognize themselves as victims of ethnic-cultural aggressions do not usually recognize themselves as victims of the homologous customs seen in personal aggression (e.g., McKenney et al., 2006; Verkuyten and Thijs, 2006). Differences in the students’ moral evaluation of both types of aggressions have been identified, indicating that inter-group ethnic-cultural aggression is more harmful and rejective than exclusively interpersonal aggression (Strohmeier et al., 2005; Monks et al., 2008). Different relationships and effects of ethnic-cultural victimization have been described in contrast to those described in personal victimization, regarding variables such as personal self-esteem, cultural self-esteem and welfare feelings, among others. For instance, it is known that personal victimization determines personal self-esteem decline (Verkuyten and Thijs, 2006), an effect that is increased when ethnic-cultural victimization also occurs, and which is mediated by the ethnic-cultural self-esteem decline (Rodríguez-Hidalgo et al., 2015). Ethnic-cultural victimization predicts the loss of a sense of pride of belonging to the ethnic-cultural group of origin, a fact which, however, does not predict personal victimization.

Ethnic-cultural victimization is being documented by the study of the victims’ voice, since it seems that in all the cases the victim recognizes that the suffered discriminatory aggression is related to him/her belonging to a different cultural group. Nevertheless, the fact of whether the aggressor recognizes and assumes reasons of ethnic-cultural difference for his/her aggressive behavior has not been studied in depth. The lack of studies in this field can be explained due to the lack of instruments sensitive to their registration. The advance in our knowledge about ethnic-cultural aggression and its integration with the knowledge on ethnic-cultural victimization would be necessary to improve our understanding of the possible ethnic-cultural bullying. The present study aims to explore whether minors who are considered aggressors of other classmates by means of ethnic-cultural discriminatory behaviors that are persistent in time recognize such motivation. We aim to know whether there is specific ethnic-cultural bullying in which the dominance-submission dynamic and the immorality included in the psychosocial-nature of bullying are recreated. For this purpose, we need to have a measurement instrument that registers such dynamic interaction of at least the two main roles of bullying — aggression and victimization — produced and suffered by girls and boys who perceived themselves as involved.

The last two decades have been especially prolific regarding the development of studies directed to obtain scales of social discrimination and different ways of exclusion. Although most of these scales have shown good psychometric properties (Bastos et al., 2010), their target population is adults and they measure by means of self-reports the subjective perception of the potential victims. This limits its use in studies on high-schoolers to some extent. In order to overcome these limitations, some researchers (Benner and Graham, 2013; Benner and Wang, 2017) have used the *Adolescent Discrimination Distress Index* registering by means of 3 of its 11 items the discrimination among high-schoolers in the last 6 months by answering *yes* or *no* (e.g., “other kids exclude you from their activities because of your race/ethnicity”). Recently, Baysu et al. (2016) used an adaptation of the *exclusion/rejection* subscale of the *Perceived Ethnic Discrimination Questionnaire*. They proposed six items in this scale which correspond to ways of victimization that high-schoolers can suffer at the hands of their peers, such as threats, exclusion or insults. For this part, Priest et al. (2014) have developed a scale for racial discrimination that takes into consideration two dimensions: (1) *direct experiences*, suffered at the hands of both other students and teachers; and (2) *vicarious experiences* toward other students. Nevertheless, developing scales of discrimination among peers which are sensitive not only to ethnic-cultural victimization but also to ethnic-cultural aggression is still a pending task. According to Bastos et al. (2010), this will allow: (a) to address the survey respondents as potential victims and as potential discrimination aggressors; and (b) to study the possible effects of frequent discrimination on health.

Furthermore, the line of studies about discriminatory, racial and/or xenophobic bullying has preferentially focused on victimization, without paying attention to ethnic-cultural aggression. The measurement instruments used in research about ethnic-cultural victimization (e.g., Verkuyten and Thijs, 2002, 2006; McKenney et al., 2006; Rodríguez-Hidalgo et al., 2015) come to a large extent from the *Olweus Bullying Victimization Questionnaire* —OBVQ— (Olweus, 1996; Solberg and Olweus, 2003). Most of these instruments are adapted from an explanation about the phenomenon of ethnic-cultural victimization in which it is clearly stated that the reason and the way of suffered aggression is based on the differences in skin color, country of origin, culture and/or religion. They provide some examples and after, they pose some referring questions. In all the cases, questions about the frequency of having been targeted with ethnic-cultural aggressions are considered, which are answered by choosing a value in the Likert-type scale. For his part, McKenney et al. (2006) posed a single general question about the frequency of being attacked in an ethnic-cultural way. In their research, Verkuyten and Thijs (2002, 2006) used four questions to register the frequency of ethnic victimization regarding several aspects: the insult and/or racial nomination within the school; the insult and/or racial nomination within neighborhood; the direct social exclusion of taking part in games due to ethnic differences within the school; and the direct social exclusion of games due to ethnic differences within the neighborhood. Shortly after, Monks et al. (2008) and Rodríguez-Hidalgo et al. (2015) studied several questions about the frequency of ethnic-cultural victimization in three different ways: direct verbal (racial insult/nomination or threat), direct relational (social exclusion) and indirect relational (rumors and/or lies spread by third parties). Recently, Rodríguez-Hidalgo et al. (2014) proposed a variation, starting from a question about the frequency of having been a victim of ethnic-cultural aggressions by classmates. Later they asked how they had been attacked and offered them a list of nine options, including physical, verbal and relational forms.

The research on personal bullying — not specifically ethnic-cultural bullying — in the last 5 years has provided us with a number of questionnaires which allow us to measure this phenomenon in a more concise and complex way, with better psychometric properties than the ones developed throughout the last decades. The OBVQ (Olweus, 1996; Solberg and Olweus, 2003) and many of its derivative questionnaires have evidenced certain limitations to register aggression (Chen et al., 2012; Ortega-Ruiz et al., 2016). The *European Bullying Intervention Project Questionnaire* —EBIPQ— (Brighi et al., 2012; Ortega-Ruiz et al., 2016) allows us to register the two dimensions of the phenomenon of bullying — victimization and aggression — and has shown excellent psychometric properties, validity and strength. The EBIPQ is sensitive to multiple aggressive behaviors that can be shown — aggression — and to multiple behaviors that can be suffered — victimization — in bullying: physical, verbal and relational, both direct and indirect. Nevertheless, this instrument does not gather any ethnic-cultural aspects, thus its sensitivity to register ethnic-cultural aggression and victimization is very low.

As it has been noted, the ethnic-cultural victimization among peers that minority groups suffer may cause: mistrust toward the majority group; disconnection with the school and a low academic performance; underestimation and disdain toward what the group of origin can contribute; and health problems. This way, the existence of ethnic-cultural bullying in a pluricultural school could potentially promote alienation, exclusion and discrimination. However, as stated before, the knowledge on ethnic-cultural bullying is still unclear at some points, most of them due to methodological issues of its study. The vast majority of studies have been carried out on small multicultural samples or on specific samples of a particular ethnic-cultural group (Goldweber et al., 2013). It is necessary to develop studies on bullying with wider representative samples in which the ethnic-cultural diversity is controlled (Lund and Ross, 2017). Moreover, most of the studies have been carried out starting from the victims’ perception: students who are the target of aggressive behaviors that can be racist, xenophobic and/or discriminatory in terms of ethnic-cultural differences. Therefore, in the context of the possible ethnic-cultural bullying, we know more about victimization. However, we do not know so much about ethnic-cultural aggression.

The present research has carried out a comparative study between personal bullying — not specifically discriminatory, racial, or xenophobic — and ethnic-cultural bullying, searching for evidence of contrast and singularity of the second in relation to the first. Its approach aims to overcome the limitations that have already been described in previous studies. Our sample is composed by a wide, representative pluricultural sample: the student population in Spain. Our first objective is to adapt and to validate the EBIPQ instrument (Brighi et al., 2012; Ortega-Ruiz et al., 2016) to measure ethnic-cultural bullying in all its dimensions of ethnic-cultural aggression and ethnic-cultural victimization. Following the shortage of scientific literature regarding validated and reliable instruments to measure ethnic-cultural bullying in all its possible dimensions of aggression and victimization, the EBIPQ could be considered as a model to develop an appropriate measurement instrument of this discriminatory abuse among peers. If we carried out an adaptation of it, its thorough and wide register of different victimization and aggression behaviors, we could use it to register exclusively those behaviors of ethnic-cultural victimization and ethnic-cultural aggression. In case of obtaining useful results, the EBIPQ-Ethnic-Cultural Discrimination Version (henceforth EBIPQ-ECD) would be at our disposal. Our initial hypothesis is that the EBIPQ-ECD would show a two-factor structure with optimal psychometric properties, which would correspond to the predominance of victimization and aggression as two well-defined factors of the phenomenon: ethnic-cultural victimization and ethnic-cultural aggression. If this hypothesis was verified, we would have at our disposal an instrument that would help progress in the study of the ethnic-cultural bullying in its two possible dimensions. The second objective is to find out if, in addition to the role of ethnic-cultural victim, the roles of the ethnic-cultural aggressor, ethnic-cultural bully/victim and non-involved are present in the phenomenon of the ethnic-cultural aggression and discrimination. The hypothesis is that the same roles of participation should be observed in both ethnic-cultural bullying and personal bullying. The third objective is to study the relationship between the involvement in personal bullying and the involvement in ethnic-cultural bullying by means of the roles of participation in order to contrast the third hypothesis. The involvement in personal bullying is expected to be different from the involvement in ethnic-cultural bullying following the participation of the different roles.

## Materials and Methods

### Participants

The subjects were selected using a random cluster sampling among all the public schools that taught Secondary Obligatory Education in Spain. We got in contact with more than 1000 educational centers that were selected randomly. Finally, 25% of these centers took part in the study. A total of 36859 students were surveyed for the research. 9490 participants were excluded from the total due to the fact that their answers were inconsistent (6250) or because we had too many subjects within clusters (3240). A final sample of 27369 Secondary Education students participated in this research (51.3% girls), aged from 11 to 18 years old, *M* = 13.94 (*SD* = 1.39), who were from the 17 autonomous communities and the 2 autonomous cities that form Spain. 630 subjects (2.3% of the total participants) did not want to reveal their cultural origin (of them or of one of their parents). Those who did reveal their cultural origin from the 26739 participants are: 79.60% Spanish natives (*n* = 21284; *M*_age_ = 13.88; *SD* = 1.37; 51% girls); 11.19% immigrants (*n* = 2992; *M*_age_ = 14.44; *SD* = 1.46; 52.4% girls); 9.21% born in Spain but whose parents are immigrants (*n* = 2463; *M*_age_ = 13.84; *SD* = 1.43; 52.1% girls). Furthermore, 334 subjects, in addition to belonging to any of the previous groups, belonged to the Roma ethnic group (*M*_age_ = 13.98; *SD* = 1.39; 41.6% girls). The main countries of origin of the participants that were not born in Spain were: 504 from Romania, 426 from Morocco, 282 from Colombia, 268 from Ecuador, 118 from Bolivia, 106 from Argentina, 99 from Dominican Republic, 98 from Bulgaria, and 80 from Peru; 1011 were from other countries.

### Instruments

We used the Spanish version of the *European Bullying Intervention Project Questionnaire* (EBIPQ) (Ortega-Ruiz et al., 2016) that begins with an explanation about how to complete it. This way, it contextualizes the participants about the possible experiences related to bullying in their environment of school, friends and acquaintances and it poses them the following question: “*Have you ever experienced any of the following situations in the last 2 months?*” To complete it, there are 14 Likert-type items with five options of response in terms of the frequency with which the different behaviors described in the items may occur, which go from “*never*” until “*yes, more than once a week.*” This questionnaire presents two subscales: victimization and aggression. Each of these subscales has got seven items, which register the same behaviors in parallel, with the difference that they are received aggression behaviors in victimization (e.g., *someone has insulted me; I have been excluded, isolated or ignored by other people;* among others) and they are expressed behaviors toward other(s) in aggression (e.g., *I have insulted and said offensive words to someone; I have excluded, isolated or ignored to someone*; among others). This questionnaire presents acceptable levels of reliability (α_total_ = 0.859; α_victimization_ = 0.826; α_aggression_ = 0.821).

We used an adaptation of the EBIPQ to the EBIPQ-ECD to measure ethnic-cultural bullying (see Appendix). This adaptation presents the same 14 original items of the EBIPQ and the same method of response (Likert-type) to measure the expressed aggressive behaviors and the received aggressive behaviors. Nevertheless, the introductory explanation that helps the participants when answering is changed in the new version. In this new version, the participants are contextualized and asked in the following way: *Now we ask you about your* ***possible experiences of discrimination*** *within your environment (school, friends, acquaintances)*, ***due to your skin color, your country of origin, your culture or your religion*** *in the last 2 months.* Thanks to this adaptation, the participants can show not only the received behaviors of ethnic-cultural aggression, but also the expressed behaviors of ethnic-cultural aggression. This adapted questionnaire presents acceptable levels of reliability (α_total_ = 0.886; α_victimization_ = 0.860; α_aggression_ = 0.851).

We used the *Cuestionario de Violencia Escolar – 3 versión Educación Secundaria Obligatoria* —School Violence Questionnaire – 3 version Secondary Obligatory Education, CUVE^3^-ESO — (Álvarez-García et al., 2012; BÁlvarez-García et al., 2013); in particular, we used two of its subscales: those that refer to the verbal violence of the students toward teachers (three items) and to the violence of teachers toward students (10 items) in order to verify the divergent validity of the main instrument. Both subscales are Likert-type and presented five options of response from 1 = *Never* to 5 = *Always*. Both subscales showed optimal values of reliability (_αV erbal violence of the students toward teachers_ = 0.837; α_V iolence of teachers toward students_ = 0.906).

### Procedure

Once the participating schools of the study were selected randomly, we got in touch with them by telephone, explaining the reason for the research and asking them if they wanted to take part in it. If the answer was negative, the next school was asked. If the answer was affirmative, they were sent a unique code of access for the school, the link to the online questionnaire, the questionnaire in paper, a letter of passive and active consent for the students’ parents and the instructions to apply the questionnaire correctly. Each educational school decided what type of letter they would give the students’ parents in order to get their consent. The teachers were those who chose when the students filled in the questionnaire within the established period of 1 month. The students invested one lesson (50 min) in order to fill in the questionnaire. At the beginning, they were indicated that the questionnaire was anonymous and voluntary. All the questionnaires were filled in online. The procedure was approved by the ethic committee of the University of Córdoba.

### Analysis

First, the structural validation of the EBIPQ-ECD scale was carried out by means of a confirmatory factorial analysis (CFA). We used the estimation method called maximum likelihood (ML), adopting the robust correction and using polychoric correlations, given the categorical nature of the variables (Flora and Curran, 2004). In order to assess the suitability, we used the Satorra-Bentler scaled chi-square (χ2S-B), chi-square divided by degrees of freedom (χ2S-B/df) (≤5 acceptable, ≤3 optimal), the Comparative Fit Index (CFI) and the Non-Normed Fit Index (NNFI) (whose values have to be ≥ 0.95). The root mean square error of approximation (RMSEA) (≤0.05) and the standardized root mean-square residual (SRMR) (≤0.08 acceptable; ≤0.05 optimal) were also considered (Hu and Bentler, 1999). The EQS 6.2 program was used for these analyses. Afterward, a Spearman’s correlation was carried out using the EBIPQ-ECD scale and the CUVE^3^-ESO subscales in order to measure the divergent validity of the instrument.

Second, descriptive analyses of frequencies of both scales were carried out. Finally, following the same theoretical criteria proposed by the authors (Del Rey et al., 2015), the prevalence and involvement in different roles were calculated in both phenomena —traditional bullying and ethnic-cultural bullying— distinguishing among non-involved, victims, aggressors, and victimized aggressors.

## Results

**Figure 1:**
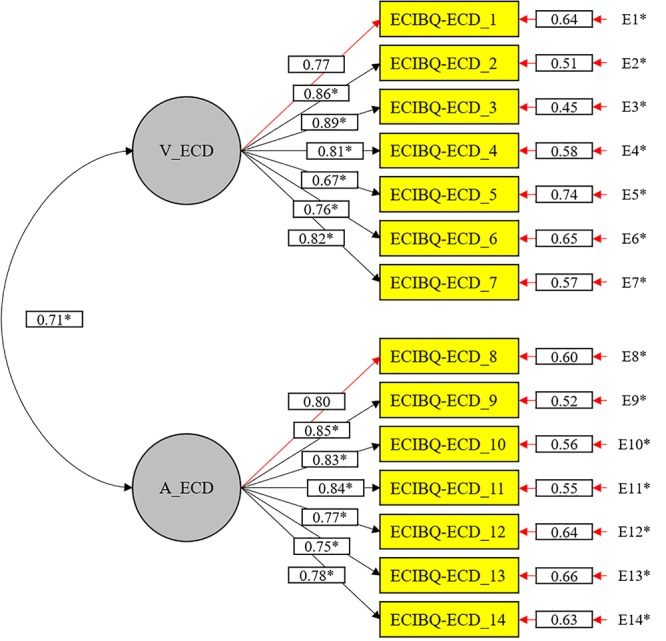
CFA of the EBIPQ-ECD scale (^∗^*p* < 0.05).

The CFA was carried out where the factorial solution of the questionnaire was subjected to contrast. The random maximum likelihood estimation (RML) was used bearing in mind the multivariate distribution that obtains a Mardia’s coefficient of 3662.25 and an estimated normality of 2366.01. The obtained results showed an optimal fitting of the solution of two dimensions that the original scale of traditional bullying presents (see Figure 1 and Table 1), with fitting index values of χ^2^ S-B = 10320.64; *p* = 0.00; RMSEA = 0.077; SRMR = 0.06; CFI = 0.96; NNFI = 0.95. The polychoric correlations show acceptable correlations that range from 0.373 to 0.784 between the variables that are part of the scale (see Table 2).

**Table 1:** Fitting indexes of the scale with different factorial answers.

Model	χ^2^ S-B	*p*	RMSEA	SRMR	CFI	NNFI
(1) Factor	22741.27	0.00	0.11	0.10	0.92	0.90
(2) Factors	10320.64	0.00	0.07	0.06	0.96	0.95
(3) Factors	28065.36	0.00	0.12	0.15	0.89	0.84
(4) Factors	30652.56	0.00	0.16	0.17	0.79	0.78

**Table 2:** Polychoric correlations – EBIPQ-ECD.

	V1	V2	V3	V4	V5	V6	V7	V8	V9	V10	V11	V12	V13	V14
**V1**	1													
**V2**	0.742	1												
**V3**	0.618	0.784	1											
**V4**	0.700	0.700	0.688	1										
**V5**	0.574	0.553	0.551	0.596	1									
**V6**	0.576	0.629	0.692	0.630	0.535	1								
**V7**	0.526	0.653	0.833	0.628	0.517	0.652	1							
**V8**	0.647	0.514	0.418	0.512	0.440	0.334	0.390	1						
**V9**	0.514	0.611	0.537	0.489	0.413	0.365	0.491	0.753	1					
**V10**	0.404	0.498	0.596	0.448	0.393	0.397	0.547	0.577	0.754	1				
**V11**	0.459	0.449	0.448	0.645	0.406	0.373	0.442	0.762	0.712	0.624	1			
**V12**	0.437	0.403	0.405	0.466	0.607	0.379	0.392	0.640	0.596	0.605	0.688	1		
**V13**	0.374	0.394	0.446	0.430	0.382	0.491	0.442	0.543	0.604	0.650	0.615	0.618	1	
**V14**	0.384	0.446	0.538	0.442	0.405	0.393	0.601	0.508	0.607	0.751	0.618	0.631	0.667	1

Afterward, in order to verify the discriminant validity of the questionnaire of bullying due to ethnic-cultural reasons, we carried out the analysis of Spearman’s rho bivariate correlation with the variables of traditional bullying (see Table 3). The results show a significant correlation in all the dimensions, which emphasizes the relationship of both phenomena but with differences since the correlations are not excessively high.

**Table 3:** Spearman’s rho – Traditional and ethnic-cultural bullying.

	1	2	3	4
(1) Traditional victimization	1			
(2) Traditional aggression	0.545^∗∗^	1		
(3) Ethnic-cultural victimization	0.719^∗∗^	0.441^∗∗^	1	
(4) Ethnic-cultural aggression	0.423^∗∗^	0.735^∗∗^	0.577^∗∗^	1

^∗∗^*The correlation is significant at the 0.01 level (two-tailed)*.

To verify the convergent validity of the questionnaire of bullying due to ethnic-cultural reasons, we carried out the analysis of Spearman’s rho bivariate correlation with the variables of school violence, where the relationship of the studied phenomenon with other dimensions of school violence and coexistence problems can be observed (see Table 4). Although the correlations are statistically significant, the low or moderate values show that both phenomena are not the same despite being related.

**Table 4:** Spearman’s rho – ethnic-cultural bullying and CUVE^3^-ESO subscales.

	1	2	3	4
(1) Ethnic-cultural victimization	1			
(2) Ethnic-cultural aggression	0.577^∗∗^	1		
(3) Verbal violence from students toward teachers	0.180^∗∗^	0.165^∗∗^	1	
(4) Violence from teachers toward students	0.195^∗∗^	0.252^∗∗^	0.450^∗∗^	1

^∗∗^*The correlation is significant at the 0.01 level (two-tailed)*.

In order to address the second objective, we first carried out an analysis of frequency of involvement in each of the roles of involvement in traditional bullying and in ethnic-cultural bullying. The data show that — from the 21732 validated responses in both questionnaires — 20.5% (*n* = 4445) of the sample were involved in traditional bullying as victims, 5.5% (*n* = 1197) as aggressors and 13.8% (*n* = 2990) in the double role of bully/victim. In the case of ethnic-cultural bullying, the percentages of involvement are lower: 12.9% (*n* = 2813) called themselves as victims, 3.8% (*n* = 815) as aggressors and 7.7% (*n* = 1676) as bully/victims.

In order to know more about the overlap between the different ways of bullying, we opted for carrying out a contingency table with the roles of one and the other. There was an unequal proportion in the value distribution, making evident the fact that there was a strong continuity between the role adopted in traditional bullying and the one adopted in ethnic-cultural bullying [χ^2^(9,21732) = 20000.731, *p* < 0.001], although with some interesting nuances (see Table 5). From the non-involved participants in ethnic-cultural bullying, 21.8% considered that they were involved in traditional bullying. From the victims of ethnic-cultural bullying: 5.2% did not consider that they were involved in traditional bullying; 20.7% were involved in the double role of bully/victim in traditional bullying; and 1.2% were involved in aggression on traditional bullying. The aggressors in ethnic-cultural bullying showed the same pattern, but the percentages were higher in the other roles. Regarding the bully/victims in ethnic-cultural bullying, most of them (85.5%) were in the same role in traditional bullying, and only a very few (2.1%) considered themselves as non-involved.

**Table 5:** Overlap between traditional bullying and ethnic-cultural bullying.

			Role in ethnic-cultural bullying				Total
			Non-involved	Victim	Aggressor	Bully/victim	
Role in traditional bullying	Non-involved	% row	98.1	1.1	0.5	0.3	100.0
		
		% column	78.2	5.2	8.5	2.1	60.3
		
		ASR	95.1	-64.0	-30.8	-50.6	
	
	Victim	% row	50.1	46.2	0.8	3.0	100.0
		
		% column	13.6	73.0	4.2	7.9	20.5
		
		ASR	-44.4	74.0	-11.7	-13.3	
	
	Aggressor	% row	50.5	2.8	40.4	6.3	100.0
		
		% column	3.7	1.2	59.4	4.5	5.5
		
		ASR	-20.8	-10.8	68.7	-1.9	
	
	Bully/victim	% row	25.0	19.4	7.6	47.9	100.0
		
		% column	4.6	20.7	28.0	85.5	13.8
		
		ASR	-69.3	11.4	12.0	88.8	

Total		% row	75.6	12.9	3.8	7.7	100.0
		
		% column	100.0	100.0	100.0	100.0	100.0

*ASR, adjusted standardized residuals*.

The corrected categorized residuals show the existence of a high overlap and maintenance of the role of the non-involved and the victims in both ways of bullying. However, within the bully/victims in traditional bullying, there are anticipated frequencies higher in the roles of victim, aggressor, and bully/victim of ethnic-cultural bullying. This indicates that this role is more spread, and that the person involved as bully/victim in traditional bullying can take the role of aggressor or victim when the ethnic-cultural nature is included.

## Discussion and Conclusion

The first objective of this research was to validate the adaptation of the original measurement instrument EBIPQ of the personal bullying to create the EBIPQ-Ethnic-Cultural Discrimination Version. By adapting the questionnaire, good fitting indexes, optimal values and a good internal consistency were obtained. In the EBIPQ-ECD, the analysis confirmed the same structure in both factors — victimization and aggression — as the original instrument showed, so the initial hypothesis was corroborated. Therefore, there is a parallelism in the measurement of the EBIPQ on the victimization and aggression behaviors, in contrast with the measurement of the EBIPQ-ECD on the discriminatory behaviors of ethnic-cultural victimization and ethnic-cultural aggression.

Regarding the second objective, the same roles of participation in the personal bullying were observed in the ethnic-cultural bullying: victim, aggressor, bully/victim, and non-involved. The second hypothesis of the study was corroborated. This evidences the subjective recognition from one part of the adolescents of being ethnic-cultural victims and/or of being ethnic-cultural aggressors. The knowledge provided regarding ethnic-cultural aggression is especially novel. There are adolescents who recognize themselves as pure ethnic-cultural aggressors. The study also reveals the existence of a mixed role of the ethnic-cultural bully/victim. We refer to adolescents who recognize themselves as a persistent object of discriminatory, racial and/or xenophobic aggressions, who at the same time recognize themselves as reiterated aggressors of the same type toward other peers because they belong to different ethnic-cultural groups. This type of mixed role has been documented in bullying (e.g., Ortega-Ruiz et al., 2016), but no scientific literature has been found to evidence its presence in the ethnic-cultural bullying. Having as an example the knowledge of the dynamic of personal bullying, we could infer that, among ethnic-cultural bully/victims we may find provocative victims, victims who start to respond aggressively and aggressors who begin to be victimized, among others, always in a dynamic of discriminatory behaviors among ethnic-cultural groups. The scientific literature has shown that these discriminatory behaviors are not only expressed and received among members of different ethnic-cultural groups, but also among members of the same group, as there are cultural subgroups such as the Hindu caste system (Eslea and Mukhtar, 2000). It is also known that adolescents from a cultural majority in a country can also be object of ethnic-cultural victimization at the hands of peers from minority groups. This is an uncommon fact, that is more likely to occur when, in the specific scholar context, the presence of the minority is higher than the prevalence of the cultural majority of the country (Monks et al., 2008).

In order to achieve the third objective, the study is based on different results. The analysis of discriminant validity between factors of the EBIPQ and the EBIPQ-ECD allows us to conclude that, although there is some overlap between personal bullying and ethnic-cultural bullying, there are also certain contrasts between both phenomena. In bullying, multiple aggression and victimization behaviors are observed. The same occurs in the case of ethnic-cultural bullying, where we observe multiple aggression and victimization behaviors that can be similar but with the distinctive characteristic that they are used to discriminate and/or used in a discriminatory way following the different ethnic origin and/or the cultural differences. More evidence of this contrast between both phenomena has been shown by comparing the roles of participation identified in each of them. Even though part of the victims, aggressors, bully/victims and those non-involved in ethnic-cultural bullying also recognize themselves in the homologous role of personal bullying, in each of these roles of ethnic-cultural bullying there’s also a considerable part who recognize themselves in a different role regarding personal bullying. This corroborates the third hypothesis of the study. Bearing in mind the observations of co-occurrence on one part and of no co-occurrence on the other between personal bullying and ethnic-cultural bullying, a possible explanation can be proposed: high-schoolers consider both phenomena as well-differentiated from the other, and the partial overlap between both is due to the contagion and/or dynamism between them. This attempt of explanation supported in the obtained results is realistic bearing in mind some of the previous evidence. It is known that in the comparison between some ways of personal victimization such as the direct verbal ways and the social exclusion, in contrast with its homologous ways of ethnic-cultural victimization, a reasonable number of students recognize themselves as victims of the ethnic-cultural forms and do not recognize themselves as victims of the personal forms (e.g., McKenney et al., 2006; Verkuyten and Thijs, 2006; Monks et al., 2008). Another important issue is that students do differentiate ethnic-cultural victimization from personal victimization well, assigning a more discriminatory, aggressive and immoral nature to the first one than to the second one (e.g., Strohmeier et al., 2005). We should also consider that ethnic-cultural victimization has shown different relationships and effects than personal victimization in some studies regarding personal self-esteem, cultural self-esteem and welfare feelings (i.e., Verkuyten and Thijs, 2002, 2006; Rodríguez-Hidalgo et al., 2015).

The contribution of the present study is far from the idea that ethnic-cultural discriminatory behaviors among peers are another way of behavior, among others, which cause victimization in personal bullying (e.g., Collins et al., 2004; Strohmeier et al., 2011). Based on the collection of knowledge about ethnic-cultural victimization (e.g., Verkuyten and Thijs, 2002, 2006; McKenney et al., 2006; Monks et al., 2008; Rodríguez-Hidalgo et al., 2014) and following the findings obtained from the present study, ethnic-cultural bullying can be described as a phenomenon of interpersonal violence that presents a discriminatory, racist and/or xenophobic nature among students, caused by intentional and reiterated aggressions throughout time, which occur in a situation of power imbalance among aggressors and victims; where both victims and aggressors recognize the ethnic-cultural motivation. The present study increases the evidence of recognition by minors who recognize themselves as systematic authors of discriminatory aggressions toward their peers based on cultural differences: ethnic-cultural aggressors and ethnic-cultural bully/victims.

Beyond the perception of subtle discriminatory behaviors in the social environment of development (Dávila, 2015), the present research shows that discrimination takes shape within the school context by means of multiple ways of aggression that are evident and cause ethnic-cultural bullying. Like in other previous studies, ethnic-cultural multivictimization has been observed to appear (Rodríguez-Hidalgo et al., 2014); even though such observation is broadened by considering the aggressors’ point of view: ethnic-cultural multiaggression is also observed.

From the findings, we get certain inferences for research, education and social promotion. Regarding the research on this field, we can conclude that the EBIPQ presents certain limitations to register aggressions and victimization typical of ethnic-cultural bullying. Other available questionnaires to measure personal bullying also present the same limitation. Therefore, the EBIPQ-ECD is proposed as a measurement instrument of ethnic-cultural bullying with certain guarantees derived from the solid experience of international and intercultural validity and quality of its direct model: the EBIPQ (Brighi et al., 2012; Ortega-Ruiz et al., 2016; Herrera-López et al., 2017). The EBIPQ-ECD allows us to register thoroughly the multiple ways of ethnic-cultural aggression and victimization and, by extension, the global evaluation of ethnic-cultural bullying. As it has been shown, this instrument is useful in order to distinguish the different roles of participation in this phenomenon. By being based on a scale and by contemplating the dimensions of aggression and victimization, unlike the previous measurement instruments of ethnic-cultural victimization (e.g., Verkuyten and Thijs, 2002, 2006; McKenney et al., 2006; Rodríguez-Hidalgo et al., 2015) and the perception of discrimination among students (e.g., Benner and Graham, 2013; Priest et al., 2014; Baysu et al., 2016; Benner and Wang, 2017), it offers great possibilities to study ethnic-cultural bullying regarding its antecedents and protective factors, as well as for its consequences. The reliable measures offered by the EBIPQ-ECD could be introduced in structural equation models. This supposes a great opportunity in the research to analyze and understand the relationships between the ethnic-cultural bullying and other aspects and phenomena.

Eradicating discrimination, racism and xenophobia at a social level entails preventing and mitigating ethnic-cultural bullying in schools. For education and to improve social promotion in pluricultural contexts, the use of the EBIPQ-ECD in minors could be very helpful to detect discriminatory aggressions and ethnic-cultural victimization. The early detection of discriminatory aggressions is essential to avoid the consolidation of ethnic-cultural bullying. If ethnic-cultural bullying is consistent in some cases, its detection will be fundamental to design and develop strategies to eliminate it.

The distinctive importance given to the ethnic-cultural bullying in comparison to the personal bullying, in its dimensions of aggression and victimization, might require reconsidering educational policies and strategies addressing the prevention and eradication of interpersonal violence and racism among students in pluricultural contexts. It would be advisable that both programs of prevention of personal bullying and programs of promotion of coexistence, such as the ones of the prevention of racism and xenophobia and of intercultural education, would bear in mind the nature of the ethnic-cultural bullying and that would dedicate specific strategies to it. Particularly in Spain, the legislated protocols of action when facing bullying do not consider the typology of ethnic-cultural bullying. It would be advisable that the protocols for these situations of abuse among peers would consider this special type and specific measurements regarding its singularity.

The limitations of the present study are associated with the use and application of self-reports as the only measurement to register information. Despite the strong point of its wide and representative sample, for future research, it would be recommendable to carry out longitudinal studies and to combine the data collection by means of self-reports with hetero-reports. This would allow to develop new lines of study. For instance, it would allow to evaluate the evolution of personal bullying and ethnic-cultural bullying and to contrast the hypothesis of contagion and/or dynamism between both phenomena. In the future, we intend to study ethnic-cultural bullying regarding sex, age, and different ethnic-cultural groups. For example, special attention must be devoted to Roma adolescents. The Roma ethnic-cultural group is one of the groups that suffers more discrimination and social exclusion in Europe. Suffering ethnic-cultural discrimination at school causes feelings of disengagement from the school as well as low expectations about what they can contribute (Cooper and Sánchez, 2016). Ethnic-cultural bullying could be a very adverse factor for programs and policies of educational and social inclusion whose purpose is assisting the Roma population. Taking into consideration the negative effects of ethnic-cultural discrimination and violence among minors (e.g., Garnett et al., 2014; Priest et al., 2014; Baysu et al., 2016; Cooper and Sánchez, 2016; Brüggemann and D’Arcy, 2017; Gkofa, 2017), it is essential to continue studying ethnic-cultural bullying. The scientific knowledge of this phenomenon will provide a better protection of the minors’ physical and psychological integrity in pluricultural contexts and the development of educational policies and strategies that are effective to prevent and eradicate discrimination, racism and xenophobia. These are the essential conditions to cultivate intercultural coexistence in schools, with an impact on the society.

## Author Contributions

All authors made substantial contribution to the theoretical framework, design, data collection or interpretation of this study and contributed to this article and approved its publication.

## Conflict of Interest Statement

The authors declare that the research was conducted in the absence of any commercial or financial relationships that could be construed as a potential conflict of interest.

## Appendix: EBIPQ-ECD

**Table A1:** Ahora te preguntamos sobre tus posibles experiencias de discriminación en tu entorno (centro escolar, amigos, conocidos), por tu color de piel, por tu país de procedencia, por tu cultura o por tu religión en los últimos dos meses.

(1) Alguien me ha golpeado, me ha pateado o me ha empujado por los anteriores motivos.

(2) Alguien me ha insultado por los anteriores motivos.

(3) Alguien les ha dicho a otras personas palabras ofensivas sobre mí por los anteriores motivos.

(4) Alguien me ha amenazado por los anteriores motivos.

(5) Alguien me ha robado o roto mis cosas por los anteriores motivos.

(6) He sido excluido, aislado o ignorado por otras personas por los anteriores motivos.

(7) Alguien ha difundido rumores sobre mí por los anteriores motivos.

(8) He golpeado, pateado o empujado a alguien por los anteriores motivos.

(9) He Insultado y he dicho palabras ofensivas a alguien por los anteriores motivos.

(10) He dicho a otras personas palabras ofensivas sobre alguien por los anteriores motivos.

(11) He amenazado a alguien por los anteriores motivos.

(12) He robado o estropeado algo de alguien por los anteriores motivos.

(13) He excluido, aislado o ignorado a alguien por los anteriores motivos.

(14) He difundido rumores sobre alguien por los anteriores motivos.
